# Supplementary material for: Identification of photoperiod-induced specific miRNAs in the adrenal glands of Sunite sheep (Ovis aries)
Source: Front Vet Sci. 2022 Jul 22;9:888207. doi: 10.3389/fvets.2022.888207 (PMC9354845; doi:10.3389/fvets.2022.888207)
Supplement: Supplementary file 1 [file Data_Sheet_1.ZIP › Supplementary file/miRDeep2 file.zip!/miRDeep2 file/Novel_Structure/LP42A1/Novel_205.pdf]

Provisional ID	:	NC_019460.2_8181
Score total	:	2.1
Score for star read(s)	:	-1.3
Score for read counts	:	-0.3
Score for mfe	:	2.2
Score for randfold	:	1.6
Score for cons. seed	:	
Total read count	:	11
Mature read count	:	6
Loop read count	:	0
Star read count	:	5

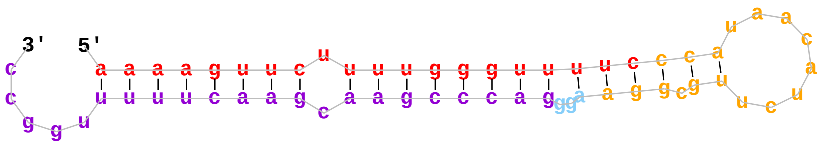

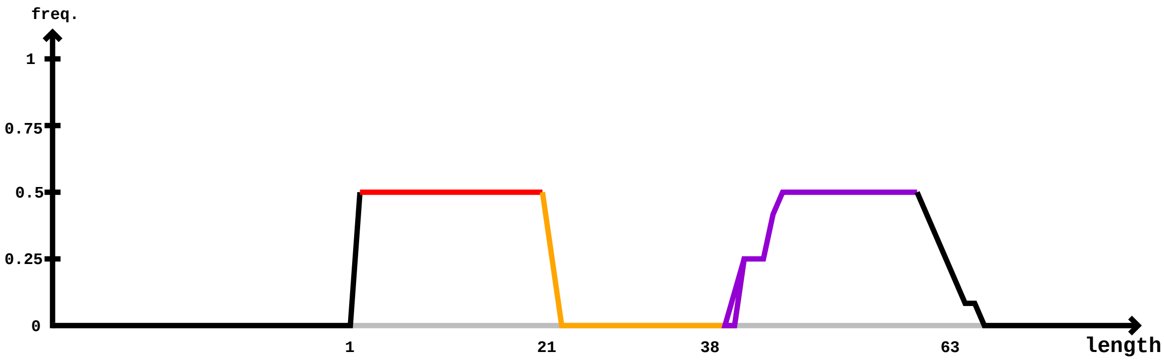


```
                                           Mature                                     Star
5'- uggccacccuuguuuuuauuggguuggcccaaaaguucuuuuggguuuuccca uaacaucuugcggaagggacccgaacgaacuuuuuggccaacccaguauuuaaucagg   -3'   obs
```

```
                              Mature                                   Star
5'- uggccacccuuguuuuuauuggguuggcccaaaaguucuuuuggguuuucccauaacaucuugcggaagggacccgaacgaacuuuuuggccaacccaguauuuaaucagg   -3'   obs
    uggccacccuuguuuuuauuggguuggcccaaaaguucuuuuggguuuucccauaacaucuugcggaagggacccgaacgaacuuuuuggccaacccaguauuuaaucagg         exp
    .......(((.(((..(((((((((((((.((((((((.((((((((((((((........)).))))..)))))))).)))))))).)))))))))))))...))).)))   reads   mm   sample
    ..............................aaaaguucGuuuggguuuuc.............................................................   6       1    seq
    ......................................................................gacccgaacgaacuuuuuggc....................   1       0    seq
    ......................................................................gacccgaacgaacuuuuuggcc...................   2       0    seq
    .........................................................................ccgaacgaacuuuuuggcca..................   1       0    seq
    .........................................................................ccgaacgaacuuuuuggccaaU................   1       1    seq
    ..........................................................................cgaacgaacuuuuuggcca..................   1       0    seq
```
